# Dissecting psychiatric spectrum disorders by generative embedding^☆^^☆☆^

**DOI:** 10.1016/j.nicl.2013.11.002

**Published:** 2013-11-16

**Authors:** Kay H. Brodersen, Lorenz Deserno, Florian Schlagenhauf, Zhihao Lin, Will D. Penny, Joachim M. Buhmann, Klaas E. Stephan

**Affiliations:** a Translational Neuromodeling Unit (TNU), Institute for Biomedical Engineering, University of Zurich & ETH Zurich, Switzerland; b Machine Learning Laboratory, Department of Computer Science, ETH Zurich, Switzerland; c Department of Psychiatry and Psychotherapy, Charité-Universitätsmedizin Berlin, Germany; d Max Planck Institute for Cognitive and Brain Sciences, Leipzig, Germany; e Wellcome Trust Centre for Neuroimaging, University College London, United Kingdom; f Laboratory for Social and Neural Systems Research (SNS), University of Zurich, Switzerland

**Keywords:** Clustering, Clinical validation, Balanced purity, Schizophrenia, Variational Bayes

## Abstract

This proof-of-concept study examines the feasibility of defining subgroups in psychiatric spectrum disorders by generative embedding, using dynamical system models which infer neuronal circuit mechanisms from neuroimaging data. To this end, we re-analysed an fMRI dataset of 41 patients diagnosed with schizophrenia and 42 healthy controls performing a numerical *n*-back working-memory task. In our generative-embedding approach, we used parameter estimates from a dynamic causal model (DCM) of a visual–parietal–prefrontal network to define a model-based feature space for the subsequent application of supervised and unsupervised learning techniques. First, using a linear support vector machine for classification, we were able to predict individual diagnostic labels significantly more accurately (78%) from DCM-based effective connectivity estimates than from functional connectivity between (62%) or local activity within the same regions (55%). Second, an unsupervised approach based on variational Bayesian Gaussian mixture modelling provided evidence for two clusters which mapped onto patients and controls with nearly the same accuracy (71%) as the supervised approach. Finally, when restricting the analysis only to the patients, Gaussian mixture modelling suggested the existence of three patient subgroups, each of which was characterised by a different architecture of the visual–parietal–prefrontal working-memory network. Critically, even though this analysis did not have access to information about the patients' clinical symptoms, the three neurophysiologically defined subgroups mapped onto three clinically distinct subgroups, distinguished by significant differences in negative symptom severity, as assessed on the Positive and Negative Syndrome Scale (PANSS). In summary, this study provides a concrete example of how psychiatric spectrum diseases may be split into subgroups that are defined in terms of neurophysiological mechanisms specified by a generative model of network dynamics such as DCM. The results corroborate our previous findings in stroke patients that generative embedding, compared to analyses of more conventional measures such as functional connectivity or regional activity, can significantly enhance both the interpretability and performance of computational approaches to clinical classification.

## Highlights

• Proof-of-concept study for defining subgroups in psychiatric spectrum diseases.
• We re-analysed an fMRI dataset of 41 schizophrenic patients and 42 controls.
• DCM parameters were highly predictive of diagnostic status (78% accuracy).
• Unsupervised clustering of patients suggested three subgroups.
• These groups were clinically distinct and differed in their negative symptom severity.

## Graphical abstract

**Figure:**
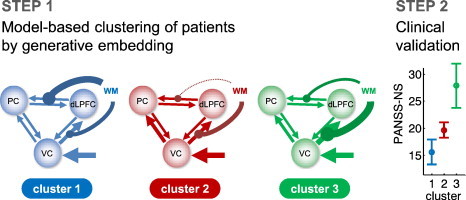

## 1 Introduction

Psychiatry has experienced a long-standing and ongoing discussion about the validity of pathophysiological concepts and clinical classification schemes. One central problem is that despite all progress in neuroscience, there has been an almost complete lack of mechanistic insights that would allow for the development of diagnostic tests for detecting pathophysiological mechanisms in individual patients. As a result, with the exception of excluding ‘external’ causes such as brain lesions or metabolic disturbances (Kapur et al., 2012), psychiatric diagnosis still relies on symptom-based definitions of disease, such as the classifications proposed by the Diagnostic and Statistical Manual Of Mental Disorders (DSM) or the International Classification of Diseases (ICD).

For example, despite high initial hopes, genetic tests have not entered clinical practice so far (Braff and Freedman, 2008; Tansey et al., 2012). This is not only because most diseases appear to be highly polygenetic, with each candidate polymorphism possibly conveying only a modest increase in risk (International Schizophrenia Consortium, 2009) and for more than one disease (Cross-Disorder Group Of the Psychiatric Genomics Consortium, 2013). More importantly, genetic tests are impeded by the presence of strong gene–environment interactions (Caspi and Moffitt, 2006). These interactions mean that even when the genome is identical the influence of different environmental factors can lead to the occurrence of different disease mechanisms and symptoms (Dempster et al., 2011; Petronis et al., 2003).

Beyond genetics, neuroimaging is another discipline which has, so far, struggled to fulfil its promise with regard to establishing practically useful diagnostic tests for psychiatry (cf. Borgwardt et al., 2012). This is despite the fact that over the past few years, neuroimaging has seen a veritable explosion in the application to psychiatric questions.

For example, numerous studies have applied *machine-learning* techniques, such as support vector machine (SVM) classification, to structural or functional magnetic resonance imaging (MRI) data. The majority of previous studies have tried to discriminate patients with a particular DSM/ICD diagnosis from healthy controls, or to disambiguate between patients from different DSM/ICD-defined diseases (see Klöppel et al., 2012, for a recent review of the application of machine-learning methods to neuroimaging data of patients). However, for many psychiatric diseases, diagnosis with respect to DSM/ICD criteria is not the key clinical problem (with some notable exceptions, such as distinguishing between unipolar and bipolar affective psychosis in first-episode patients). Therefore, machine-learning approaches which use diagnostic labels from DSM/ICD for training a classifier applied to neuroimaging data can at best reproduce the presently established diagnostic classification, but using a considerably more expensive and complicated procedure.

Instead, it seems more fruitful to develop statistical techniques for predicting future variables which are important for clinical decision making, e.g., whether a particular patient with mild cognitive impairment will develop Alzheimer's disease within a certain period or not (Davatzikos et al., 2008; Lehmann et al., 2012). One prominent hope is that biological markers derived from neuroimaging procedures may enable more accurate predictions of treatment response or disease trajectory than the behavioural and cognitive symptoms on which current DSM/ICD diagnoses are based.

This approach is logistically considerably more challenging than the attempt of reproducing DSM-based disease definitions since it requires longitudinal studies. Nevertheless, a few recent studies have been able to demonstrate that it may be possible to predict individual treatment response (e.g., Costafreda et al., 2009; Liu et al., 2012; Szeszko et al., 2012) or clinical outcome (e.g., Koutsouleris et al., 2012; Mourao-Miranda et al., 2012; Siegle and Thompson, 2012) from structural or functional MRI data, using multivariate classification. If such a procedure could be established that allowed, with sufficient sensitivity and specificity, for clinically relevant decisions, it might indeed become a cost-effective tool for clinical decision-making. Still, however, any such approach would effectively remain a ‘black-box’ classifier, providing very limited insights, if any, into disease mechanisms. This is a fundamental limitation, since without mechanistic interpretability no diagnostic procedure can inform a change in disease concepts or guide the development of future therapies.

A potential alternative to black-box classification is to embed classification into a space spanned by the parameters of a generative model which explains how the measured data could have arisen from underlying neurophysiological mechanisms (e.g., synaptic connections between distinct neuronal populations). This is the generative-embedding approach which we recently introduced to neuroimaging (Brodersen et al., 2011a).

In this previous work, we demonstrated that a six-region dynamic causal model (DCM) of the early auditory system during passive speech listening could predict, with near-perfect accuracy (98%), the absence or presence of a ‘hidden’ (i.e., outside the field of view) lesion in aphasic patients compared to healthy controls. Critically, this model-based classification approach not only significantly outperformed conventional approaches, such as searchlight classification on the raw fMRI data or classification based on functional connectivity between the same regions; more importantly, it also highlighted network mechanisms which distinguished the two groups. In this case, the connections from the right to the left hemisphere were particularly informative for enabling this subject-by-subject classification, suggesting that the remote lesion prominently affected interhemispheric transfer of language information to the dominant hemisphere.

Mechanistically interpretable approaches like generative embedding have potential for significantly enhancing model-based predictions of clinically relevant variables such as outcome or treatment response. However, these approaches are of equal importance for addressing a second fundamental problem in psychiatry: the nature of psychiatric nosology itself, i.e., the disease definitions that determine clinical diagnostics and classification. As described above, DSM defines diseases purely on the basis of symptoms that can be assessed by means of structured interviews. This approach was introduced a few decades ago to ensure the reproducibility of diagnostic statements across clinicians and institutions. However, the consequence of its entirely phenomenological nature is that the resulting disease concepts are completely agnostic about underlying mechanisms. Furthermore, many empirical studies have questioned the clinical validity of this classification scheme, demonstrating problematic predictive validity with regard to treatment and outcome (e.g., Johnstone et al., 1988; Johnstone et al., 1992). It is therefore not surprising that this phenomenological definition of diseases has received substantial criticism, and alternatives are being sought, such as the Research Domain Criteria (RDoC; Insel et al., 2010) which aim to redefine psychiatric diseases based on pathophysiological mechanisms. Key challenges for this endeavour are how such pathophysiological mechanisms can be detected in the individual, and how they are combined to produce a meaningful classification.

We have previously argued that a pathophysiologically informed dissection of psychiatric spectrum diseases, such as schizophrenia, into physiologically defined subgroups should be guided by model-based estimates of synaptic physiology from neuroimaging and electrophysiological data (Stephan, 2004; Stephan et al., 2009). This approach requires modelling techniques which can be applied to non-invasive measures of brain activity in individual patients and which are capable of inferring neurophysiological mechanisms at the circuit level. One such method is dynamic causal modelling (DCM; Friston et al., 2003), a Bayesian framework for inferring neurophysiological mechanisms from neuroimaging data.

Previous electrophysiological studies have demonstrated that DCM can provide valid information on mechanisms which represent potential key dimensions of psychiatric disease, e.g., excitation–inhibition balance, synaptic plasticity by NMDA receptors, or its regulation by neuromodulatory transmitters such as dopamine or acetylcholine (Moran et al., 2011a,b; Schmidt et al., 2012). When applied to fMRI data, DCM allows for less fine-grained representation of physiological mechanisms and is largely restricted to inferring on synaptic coupling between large, undifferentiated neuronal populations. Nevertheless, even this coarse physiological representation has proven useful for distinguishing groups with different cognitive or disease states, such as: the presence vs. absence of a ‘hidden’ lesion (see above; Brodersen et al., 2011a,b); Parkinson patients on vs. off dopaminergic medication (Rowe et al., 2010); individuals with different types of synaesthesia (Van Leeuwen et al., 2011); patients suffering from depression vs. healthy controls (Desseilles et al., 2011); or congenitally blind vs. late-onset blind individuals (Collignon et al., 2013).

Using DCM as the basis for generative embedding is therefore an attractive approach for establishing clinically relevant ‘model-based assays’ that can characterise, in an unsupervised fashion, patient subgroups by differences in circuit-level mechanisms. The present study provides a first proof of concept that illustrates the plausibility of this notion in a patient sample.

In the first part of this paper, we review key concepts of generative embedding and introduce procedures that are specific for its application in an unsupervised context, i.e., model-based clustering. Keeping a clinical audience in mind, we have written this Methods section in a tutorial-like fashion wherever possible. We then present a concrete example where we examine whether schizophrenia patients can be classified into subgroups that are distinguished by different neurophysiological mechanisms underlying working memory. To this end, we used fMRI data from 41 schizophrenic patients and 42 healthy controls during a visual working-memory task which had previously been characterised by a three-region DCM of neuronal dynamics in a visual–parietal–prefrontal network (see Deserno et al., 2012, for the original analysis).

Working memory is a useful construct for testing the feasibility of model-based clustering of schizophrenia patients, given that it is a core component of cognitive control which is frequently disturbed in schizophrenia (Lee and Park, 2005). Cognitive deficits in the domain of working memory have also become a primary research target (Barch et al., 2012; Lewis and Gonzalez-Burgos, 2006) since they have been shown to be a major predictor of clinical outcome (Bowie et al., 2008; Nuechterlein et al., 2011; Shamsi et al., 2011; Vesterager et al., 2012), a predictor of the transition from an at-risk mental state to the onset of the disease (Fusar-Poli et al., 2012), and, given their presence in first-degree relatives, potentially indicative of vulnerability (Snitz et al., 2006).

Using the subject-specific posterior parameter estimates of the original model as the basis for generative embedding, we address three questions. First, does this simple three-region DCM enable above-chance classification of individual subjects, distinguishing successfully between patients and controls, and if so, how does it compare to classification based on functional connectivity and regional activity? Second, if we disregard all knowledge about the diagnostic status of our subjects, will an unsupervised clustering approach operating on the DCM parameter estimates recognise the presence of two groups, i.e., patients and controls? And finally, focusing on the patients only, is there evidence for neurophysiologically defined subgroups within the schizophrenic spectrum that are distinguished by differential network mechanisms and are related to independent clinical variables?

## 2 Methods

**Fig. 1:**
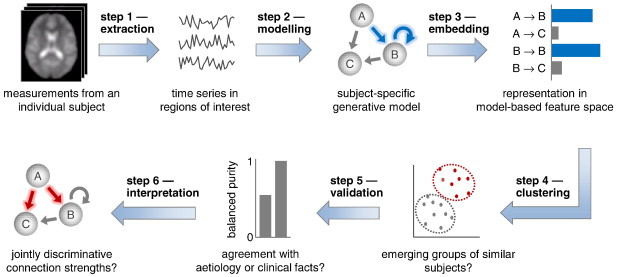
Conceptual overview of model-based clustering by generative embedding. This schematic illustrates how generative embedding enables model-based clustering of fMRI data. First, separately for each subject, BOLD time series are extracted from a number of regions of interest. Second, subject-specific time series are used to estimate the parameters of a generative model. Third, subjects are embedded in a generative score space in which each dimension represents a specific model parameter. This space implies a similarity metric under which any two subjects can be compared. Fourth, a clustering algorithm is used to identify salient substructures in the data. Fifth, the resulting clusters are validated against known external (clinical) variables. Once validated, a clustering solution can, sixth, be interpreted mechanistically in the context of the underlying generative model.

Model-based clustering by generative embedding comprises six steps: (i) extraction of time series; (ii) generative modelling and model inversion; (iii) embedding in a generative score space; (iv) clustering, including model-order selection; (v) validation with respect to independent criteria such as clinical information; and (vi) interpretation of the neurophysiological mechanisms (encoded by the parameter estimates) that define the identified subgroups. This section describes these steps in detail (Fig. 1).

### 2.1 Extraction of time series

The first step in a generative-embedding analysis concerns the extraction of data features that will be subject to modelling. In this paper, we illustrate generative embedding for fMRI data, adopting the same approach to time-series extraction as used previously in the context of model-based classification (Brodersen et al., 2011a,b). Specifically, we begin by specifying a set of regions of interest (ROIs), which could be defined anatomically or by means of a functional contrast. The choice of these regions is informed by pre-existing concepts which relate specific neuronal circuits to the expression of specific cognitive processes or behavioural symptoms. For example, the empirical application described below focuses on a visual–parietal–prefrontal network. These are regions whose involvement in visual working memory is well-established (see Rottschy et al., 2012, for a comprehensive meta-analysis of fMRI studies of working memory); in turn, working memory is a core component of cognitive control which is frequently disturbed in schizophrenia (Lee and Park, 2005). Following the definition of this network, we compute, for each region, the first eigenvariate based on all voxels contained in that region, which yields a region-specific representative time course of blood oxygen level dependent (BOLD) activity.

In order to enable an unbiased clustering analysis, it is important that the selection of time series is not based on information that is related to the differences (e.g., group membership) which we hope to disclose by clustering. For example, when assessing how well an unsupervised clustering procedure assigns patients and controls to their respective categories, between-group contrasts should not be used for the definition of regions of interest. In previous work, we analysed in detail which procedures for choice of regions and time-series extraction ensure unbiased results in analyses where generative embedding is used for model-based classification (Brodersen et al., 2011a,b). Here, we extend this analysis to model-based clustering.

**Fig. 2:**
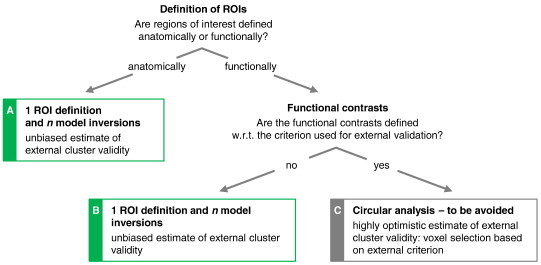
Options for network construction for generative embedding. An important design decision in model-based clustering analyses is the criterion by which regional time series are extracted from the data. One option is to define regions anatomically, followed by the separate inversion of the model for each subject. A clustering solution obtained in this way can be safely evaluated with respect to an external variable (Procedure *a*). A frequent alternative is to define regions in terms of a functional contrast. As before, models are inverted in a subject-by-subject fashion (Procedure *b*). This allows for an unbiased estimate of the external validity of the resulting clustering solution but requires, critically, that the functional contrast not be based on the same variable that is used for external validation (Procedure *c*).

As illustrated in Fig. 2, unbiased estimates of the external validity of a clustering solution can be obtained when time series are selected on the basis of anatomical contrasts (Fig. 2A) or functional contrasts that are unrelated to the variable used for external validation (Fig. 2B). By contrast, the use of the external variable itself (or a variable that is correlated with it) would introduce circularity and may easily lead to an overoptimistic estimate of cluster validity (Fig. 2C). For example, using a ‘patient–controls’ contrast for regional time-series selection should be avoided when the subsequent clustering is to be evaluated w.r.t. disease state; in such a case, it would simply not be surprising if the obtained clusters could be mapped accurately onto the groups of patients and controls. In the present study, regions of interest were therefore defined both anatomically and functionally without reference to clinical variables used for validation.

### 2.2 Generative modelling and model inversion

Generative embedding rests on the specification and inversion of a *generative model* of the data. In brief, a generative model encodes a mechanistic process of how data or measurements arise by combining a prior distribution (on parameters and/or states) with a likelihood function (Bishop, 2007). As in our previous publication (Brodersen et al., 2011a,b), we use a DCM of fMRI data (Friston et al., 2003). DCM regards the brain as a nonlinear dynamical system of neuronal populations that interact via synaptic connections, and an experiment as a designed perturbation of the system's dynamics (for a conceptual overview of DCM, see Stephan et al., 2010). While the mathematical formulation of DCMs varies across measurement types, common mechanisms modelled by all DCMs include synaptic connection strengths and experimentally induced modulation thereof (e.g., Chen et al., 2008; David et al., 2006; Daunizeau et al., 2009; Moran et al., 2009; Stephan et al., 2008). Generally, DCMs strive for neurobiological interpretability of their parameters; this is one core feature distinguishing them from most alternative approaches to modelling neuroimaging data. The physiological interpretability of DCMs makes them a particularly attractive candidate for generative embedding.

DCMs are hierarchical models with at least two layers. The first layer is a *neuronal model*, describing the dynamics of interacting neuronal populations under the influence of experimentally controlled perturbations. Its parameters are neurobiologically interpretable to some degree, representing, for example, synaptic weights and their context-specific modulation. Experimental manipulations *u* enter the model either by eliciting responses through direct influences on specific regions (e.g., sensory inputs) or by modulating the strength of coupling among regions (e.g., short-term synaptic plasticity due to task demands or learning).

In this paper, we will use the classical bilinear DCM for fMRI (Friston et al., 2003) as implemented in the software package SPM8/DCM10 (r4290). Its neuronal model rests on a deterministic ordinary differential equation

(1) $$\frac{\mathrm{dx}\left(t\right)}{\mathrm{dt}}=f\left(x\left(t\right),\theta_{n},u\left(t\right)\right)=\left(A+\sum_{j}u_{j}\left(t\right)B^{\left(j\right)}\right)x\left(t\right)+\mathrm{Cu}\left(t\right)$$

which represents a low-order approximation to any nonlinear dynamical system with known perturbations (Stephan et al., 2008). Here, *x*(*t*)  is the neuronal state vector *x* at time *t*, *A* is a matrix of endogenous (fixed) connection strengths, *B*^(*j*)^ represents the additive change of these connection strengths induced by modulatory input *u*_*j*_, and *C* denotes the strengths of direct (driving) inputs.

The second layer of a DCM is a biophysically motivated *forward model* that describes how a given neuronal state translates into a measurement. The exact form of the model depends on the measurement modality. In the case of fMRI, the haemodynamic forward model can be written as

(2) $$y\left(t\right)=g\left(x\left(t\right),\theta_{h}\right)+\epsilon$$

where *g*(⋅) is a nonlinear operator that translates a neuronal state *x*(*t*) into a predicted BOLD signal via changes in vasodilation, blood flow, blood volume, and deoxyhaemoglobin content (see Stephan et al., 2007, for details). The forward model has haemodynamic parameters *θ*_*h*_ and Gaussian measurement error *ϵ*. The haemodynamic parameters primarily serve to account for variations in neurovascular coupling across regions and subjects and are typically not of primary scientific interest. In addition, the haemodynamic parameters exhibit fairly strong inter-dependencies (high posterior covariances; Stephan et al., 2007), which makes it difficult to establish the distinct contribution afforded by each parameter. For these reasons, the model-based clustering analyses in this paper will be based exclusively on the neuronal parameters *θ*_*n*_.

DCM uses a fully Bayesian approach to parameter estimation, with physiologically informed priors for the haemodynamic parameters and zero-mean priors for the coupling parameters (Friston et al., 2003). Combining the prior density over the parameters *p*(*θ*|*m*) with the likelihood function *p*(*y*|*θ*, *m*) yields the posterior density *p*(*θ*|*y*, *m*). This inversion can be carried out efficiently by maximising a variational free-energy bound to the log model evidence, ln *p*(*y*|*m*), under Gaussian assumptions about the posterior (the Laplace assumption; see Friston et al., 2006, for details). Given *d* parameters, model inversion thus yields a subject-specific multivariate normal probability density $q\left(\theta |\hat{\mu },\hat{\sum }\right)\approx p\left(\theta |y,m\right)$ that is fully described in terms of a vector of posterior means $\;\hat{\mu }\in {\mathbb{R}}^{d}$ and a covariance matrix $\hat{\sum }\in {\mathbb{R}}^{d\times d}$. Model inversion proceeds in an unsupervised and subject-by-subject fashion, i.e., in ignorance of clinical labels that may later be used in the context of classification or clustering.

In summary, DCM provides a generative model for explaining measured time series of brain activity as the outcome of hidden dynamics in an interconnected network of neuronal populations under the influence of experimentally induced perturbations. Inverting such a model means inferring the posterior distribution of the parameters of both the neuronal and the forward model from observed responses of a specific subject. Due to its physiological interpretability and applicability to single-subject data, DCM has previously been successfully used in model-based classification (Brodersen et al., 2011a,b) and is an equally attractive candidate for model-based clustering.

### 2.3 Embedding in a generative score space

To obtain a clustering of subjects, one might think of representing each subject as a vector of voxel-wise activity over time. Such a feature space would retain all information we have measured across the whole brain. However, this is neither statistically feasible (because the number of features per subject would vastly exceed the number of subjects) nor conceptually desirable as such an overly rich representation will not only include features that are relevant for the purpose of clustering (i.e., pathophysiological processes) but also many irrelevant distractors and measurement noise which will decrease performance. This problem of feature selection poses a key research theme in machine learning and requires appropriate techniques for dimensionality reduction. One attractive option is to embed the data in a feature space that is constructed using a generative model (Lasserre et al., 2006; Martins et al., 2010; Minka, 2005; Perina et al., 2010). This feature space, referred to as a *generative score space*, embodies a model-guided dimensionality reduction of the observed data.

In the context of DCM, a straightforward way of creating a generative score space is to consider the posterior expectations of model parameters of interest (e.g., parameters encoding synaptic connection strengths). More formally, we can define a mapping ℳ_Θ_ → ℝ^*d*^ that extracts a subset of point estimates $\hat{\mu }:=\theta |x,m$ from the posterior distribution *p*(*θ*|*x*, *m*). This simple *d*-dimensional vector space represents a selective summary of network mechanisms (e.g., connection strengths between regions but not haemodynamic parameters), as opposed to activity levels within these regions. Additionally, one could incorporate elements of the posterior covariance matrix into the vector space. This would be beneficial if class differences were revealed by the precision with which connection strengths can be estimated from the data.

It is worth emphasising that, because a generative-embedding approach rests upon a mechanistically motivated dynamical systems model, a model-based feature space of the sort described above is implicitly based on a highly nonlinear mapping: from individual measurement time points to posterior parameter expectations of an underlying dynamical system. In addition, the generative score space is not just driven by the data themselves; because the generative model is inverted in a fully Bayesian fashion, the resulting space incorporates all the domain knowledge that drove the specification of prior densities over model parameters. These aspects may be critical when aiming for an interpretable clustering solution, as described next.

### 2.4 Clustering

Our approach rests on the model-guided creation of a generative score space. In principle, subsequent clustering can then be carried out in this space using any established clustering technique. However, in the context of dissecting psychiatric spectrum diseases, an important challenge is to infer on the most likely number of subgroups which are not known a priori. This advocates the use of clustering techniques that evaluate the relative goodness of different clustering solutions, for example by model selection.

A suitable technique for inferring on the number of clusters via model selection is the variational inversion of a Gaussian mixture model (GMM). In this model, the likelihood of an individual subject's data *x*_*i*_ is given by

(3) $$p\left(x_{i}|z_{i},\mu ,\Lambda \right)=\sum_{k=1}^{K}N{\left(x_{i}|\mu_{k},\Lambda_{k}^{-1}\right)}^{z_{i,k}}.$$

The above formulation corresponds to a clustering model with *K* clusters, defined in terms of means *μ*_*k*_ and precision (or inverse covariance) matrices Λ_*k*_. The latent variable *z*_*i*_ has a 1-of-*K* encoding and indicates which cluster the data *x*_*i*_ belong to:

(4) $$p\left(z_{i}|\pi \right)=\prod_{k=1}^{K}\pi_{k}^{z_{i,k}}.$$

The mixing coefficients *π* denote the relative contribution of different clusters to the data. Thus, the log likelihood of an entire dataset (of i.i.d. subjects) *X* is given by

(5) $$\ln p\left(X|Z,\mu ,\Lambda \right)=\sum_{i=1}^{n}\ln\sum_{k=1}^{K}N{\left(x_{i}|\mu_{k},\Lambda_{k}^{-1}\right)}^{z_{i,k}}.$$

One approach to estimating the parameters of the above model is via the expectation–maximisation (EM) algorithm, an iterative framework for finding maximum-likelihood parameter estimates of models with latent variables (such as cluster assignments). This approach is simple and powerful; however, it risks overfitting the data and can be prone to singularities (when a Gaussian component collapses onto a single data point, causing the log likelihood function to diverge to infinity). In addition, a maximum-likelihood formulation of a GMM assumes that the optimal number of the Gaussian components is known a priori.

To avoid the above limitations, we use a variational Bayesian approach to inverting a GMM (Attias, 2000; Bishop, 2007). This approach eschews the problem of singularities by introducing a suitable prior over the parameters (see Appendix A for details). When using this prior, the only approximating assumption required to obtain a variational solution is the mean-field assumption

(9) $$q\left(Z,\pi ,\mu ,\Lambda \right)=q\left(Z\right)q\left(\pi ,\mu ,\Lambda \right),$$

where *q*(⋅) is the desired variational approximation to the true posterior *p*(⋅|*X*). This approach also allows us to determine the optimal number of clusters by means of Bayesian model selection. Specifically, we can compute a *free-energy* bound to the log model evidence, or marginal likelihood,

(10) $$\begin{array}{l} F\left(q,X\right)\approx \ln p\left(X\right)\\ \approx \ln∭p\left(X|Z,\pi ,\mu ,\Lambda \right)p\left(Z,\pi ,\mu ,\Lambda \right)dZd\pi d\mu d\Lambda , \end{array}$$

which denotes the likelihood of a model (here: of the cluster structure) given an observed dataset X. It represents a principled measure of model goodness that trades off accuracy (model fit) against model complexity (MacKay, 1992; Penny et al., 2004). Typically, a log evidence difference higher than 3 is considered to represent strong evidence in favour of one model relative to another (Kass and Raftery, 1995); when comparing two models, this corresponds to a posterior probability of the first model, given the data, higher than 95%. In the context of our application, by comparing the (approximate) log evidence of different Gaussian mixture models, each assuming a different number of clusters, we can evaluate the relative plausibility of different cluster solutions in a principled way based on probability theory, optimizing the trade-off between accuracy (fit) and model complexity and thus preventing overfitting.

### 2.5 Validation

As explained above, the clustering solution with the highest model evidence yields the most likely substructure given the data and the GMM assumptions. However, any proposed clustering solution remains an untested hypothesis unless we explicitly validate it against a known structure that is external to the clustering model itself. We therefore have to assess explicitly how well a given clustering solution matches an external criterion (which is independent from the actual data on which clustering was performed), such as clinical status or treatment response. If the external criterion is of a categorical nature, this can be done, for instance, by computing the *purity* of the solution (e.g., Manning et al., 2008).

Informally, purity measures how homogeneous the obtained clusters are. A perfectly homogeneous cluster contains only subjects from the same class; whereas a heterogeneous cluster contains a mixture of data points from different classes. Homogeneous clusters indicate that the clustering solution has picked up the implicit grouping structure defined by the external variable (which, critically, must have been unavailable to the clustering procedure itself). To compute the purity of a solution, all data points are assigned to the class label that occurs most frequently in the associated cluster. Purity is then calculated as

(13) $$\mathrm{purity}\left(\Omega ,\mathbb{C}\right):=\frac{1}{n}\sum_{k=1}^{K}\max_{j}\left|\omega_{k}\cap c_{j}\right|,$$

where Ω = (*ω*_1_,*ω*_2_, …,*ω*_*k*_) represents the clustering solution such that *ω*_1_ contains the indices of all those subjects for which the first cluster had the highest posterior probability. Given the set of external (true) class assignments ℂ = (*c*_1_,*c*_2_, …,*c*_*j*_), the term |*ω*_*k*_ ∩ *c*_*j*_| represents the number of subjects in cluster *k* with external label *j*. Normalised by the number of subjects *n*, purity thus is a number between 0 and 1 and indicates the degree to which the obtained clustering solution agrees with grouping structure implied by an external categorical variable.

One limitation of the definition in Eq. (13) is its misleading nature when applied to imbalanced datasets where different subgroups vary in the number of subjects they contain. The underlying issue is exactly the same as with classification accuracy, which is a misleading measure of classification performance when the data are not perfectly balanced. In these cases, the *balanced accuracy* is a more useful performance measure as it removes the bias that typically arises when applying a classification algorithm to an imbalanced dataset (Brodersen et al., 2010).

Here, we introduce the same idea to provide a bias correction for the purity of a clustering solution. Specifically, we define the *balanced purity* as

(14) $$\mathrm{bp}\left(\Omega ,\mathbb{C}\right):=\left(1-\frac{1}{n}\right)\left(\frac{\mathrm{purity}\left(\Omega ,\mathbb{C}\right)-\xi }{1-\xi }\right)+\frac{1}{n^{\cdot }}.$$

In the above expression, *ξ* is the degree of imbalance in the data, defined as the fraction of subjects associated with the largest class (i.e., 0.5 ≤ *ξ* < 1). When cluster assignments perfectly agree with the external variable, the balanced purity is 1. By contrast, when cluster assignments are random, the quantity drops to 1/*K* (e.g., 0.25 if the external variable defines 4 groups). In this way, the balanced purity can be interpreted in the same way as the (balanced) accuracy of a classification algorithm: it indicates the probability with which a new subject with a particular label would be assigned to a cluster in which the majority of subjects are associated with that exact same label.

Rather than defining discrete categories, external variables may sometimes be continuous. For example, we might want to assess to what extent an obtained clustering solution is related to a (continuous) measure of clinical symptoms or outcome. In the case of a continuous external variable, the concept of purity no longer applies. Instead, we can validate a solution by attempting to reject the (null) hypothesis, using a one-way ANOVA, that the latent distribution of the external variable has the same mean in all clusters. In the next section, we will present examples of both categorical and continuous variables for external validation of a clustering solution.

## 3 Results

To demonstrate the potential utility of model-based clustering in a clinical setting, we reanalysed a previously published fMRI dataset with *n* = 83 subjects, consisting of: (i) a group of 41 patients diagnosed with schizophrenia according to DSM-IV (10 female; mean age 34.1 years; SD 10.4); and (ii) a group of 42 healthy controls (19 female; mean age 35.4; SD 12.3). A brief summary of patient demographics, the task, data acquisition, and preprocessing is provided below; we refer to Deserno et al. (2012) for more detailed information on all of these aspects.

35 patients received treatment with typical antipsychotic medication (haloperidol 22; flupenthixol 10; fluphenazine 2; perazine 1); five patients were medicated with atypical drugs (aripriprazole 2; olanzapine 2; risperidon 1); and one patient did not receive any medication. The mean medication dosage was 523.3 ± 316.3 mg (mean ± SD) in chlorpromazine equivalents. Psychopathological symptoms were assessed with the positive and negative syndrome scale (PANSS) (Kay et al., 1987), showing a mean total PANSS score of 78.9 ± 28.2 (positive symptoms: 20.2 ± 8.0; negative symptoms: 20.2 ± 8.5; general psychopathology 37.8 ± 15.0). The patients had a mean illness duration of 5.8 ± 6.8 years, a mean age of onset of 28.6 ± 8.6 years, and a mean number of 3.1 ± 3.1 episodes.

Subjects were engaged in a numeric *n*-back working-memory task with two blocked conditions: in the ‘2-back’ condition, a button press was required when the current number was identical to the number two trials ago; in the ‘0-back’ condition, a button press was required every time the number zero appeared. Each condition was presented in six blocks of 22 trials each, with three targets per block. Each number was displayed for 500 ms, separated by a 900 ms inter-stimulus interval. Task blocks were alternated with resting blocks during which only visual fixation was required.

Functional imaging data were acquired on a 1.5 T MRI scanner (Siemens Magnetom Vision) using whole-brain gradient-echo echo-planar imaging (TR 2600 ms; TE 40 ms; flip angle 90°; matrix 64 × 64; voxel size 4 × 4 × 5.5 mm^3^). Following spatial preprocessing including realignment, normalisation to the Montreal Neurological Institute (MNI) template, and smoothing using an isotropic Gaussian kernel (FWHM 8 mm), a standard mass-univariate voxel-wise analysis was performed using a general linear model (GLM), followed by DCM and Bayesian model selection (BMS); the results are described in Deserno et al. (2012).

**Fig. 3:**
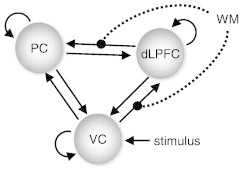
The dynamic causal model that was chosen as a basis for generative embedding. This figure summarises the structure of the three-region DCM suggested by Deserno et al. (2012). The model consists of visual, parietal, and prefrontal regions. Trial-specific visual information is modelled to provide driving input for visual cortex, whereas working-memory demands (2-back condition) are allowed to alter the strength of visual–prefrontal and prefrontal–parietal connections.

Here, we re-examined this dataset using the procedure shown in Fig. 1 and the DCM shown in Fig. 3. This DCM is the model that Deserno et al. (2012) found to have the highest evidence in the left hemisphere when pooling over healthy volunteers and patients. Note that this model comparison did not concern group discriminability but assessed how well different models explained the measured data (i.e., regional time series) per se, regardless of group membership. Regions of interest were defined anatomically and functionally without reference to any of the variables that would later be used for external validation, corresponding to the unbiased procedures shown in Fig. 2A,B.

Model inversion was carried out separately for each subject, followed by the construction of a generative score space on the basis of the posterior means of all neuronal model parameters. The resulting space contained 12 features: 6 interregional connections; 3 self-connections (*A* matrix); 2 modulatory parameters (*B* matrix); and 1 visual input parameter (*C* matrix).

Using this model-based feature space, we applied both supervised (SVM) and unsupervised (GMM) learning techniques to address the three questions outlined in the Introduction: does our rather simple three-region DCM classify patients and controls with above-chance accuracy? Second, when considering all subjects, will unsupervised clustering correctly indicate the presence of two major groups, i.e., patients and controls? And finally, focusing on the patients only, is there evidence for neurophysiologically defined subgroups within the schizophrenic spectrum?

One important subtlety is that the initial modelling results obtained by DCM might be affected by other factors than processes related to diagnostic status. We therefore regressed out *sex*, *handedness*, and *age*, using a separate multiple linear regression model for each model parameter. Thus, model-based classification was carried out on the residuals of parameter estimates after removing the potential confounds listed above. It is worth noting at this stage that neither medication nor symptom severity were considered as confounds, but as external variables of interest against which the interpretability of the proposed cluster solution was evaluated (see below).

### 3.1 Model-based classification

Before turning to model-based clustering, we initially adopted a supervised approach and used a classification algorithm to distinguish between patients and healthy controls. Following our previously published procedure of generative embedding for model-based classification (Brodersen et al., 2011a,b), we used the posterior expectations of DCM parameter estimates for training and testing a linear support vector machine as implemented in LIBSVM (Chang and Lin, 2011) on subject-specific connectivity patterns, using 5-fold cross-validation. Prior to classification, all DCM parameters were centred and standardized to have a mean of 0 and a standard deviation of 1 across subjects.

**Fig. 4:**
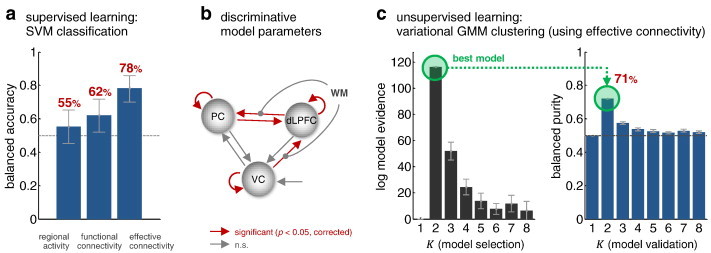
Model-based classification and clustering of all subjects. Fig. 4a shows the result of a supervised classification analysis under 5-fold cross-validation. It can be seen that schizophrenic patients can be best distinguished from healthy controls using generative embedding; this performance was significantly higher compared to classification based on functional connectivity or regional activity (see main text for details). Panel b illustrates which model parameters differed significantly between patients and controls (two-sample *t*-tests on individual model parameters with Bonferroni correction for multiple tests), thus contributing most strongly to the classification result. Panel c reports the results of an unsupervised analysis, using a variational Bayesian Gaussian mixture model that operates on DCM parameter estimates. This analysis finds the highest evidence for a model consisting of two clusters. These two clusters correspond to patient and control groups with almost the same accuracy as the supervised classification analysis (see main text for details).

The algorithm was able to separate patients and controls with a balanced accuracy of 78% (Fig. 4a). We evaluated the significance of all classification results by considering the posterior distribution of the balanced accuracy in a Bayesian beta-binomial model with a flat prior (see Brodersen et al., 2010, 2013, for details). Under a Bayesian view, significance is assessed by considering the area under the posterior density below chance, which we refer to as the posterior infraliminal probability *p*. This probability has a more intuitive interpretation than a classical *p*-value. Rather than denoting the probability of observing the data (or more extreme data) under the ‘null hypothesis’ of a chance classifier (classical *p*-value), the infraliminal probability represents the (posterior) probability that the classifier operates at or below chance (cf. Brodersen et al., 2012). For the classification result of 78% reported above, this infraliminal probability was *p* < 0.001, i.e., classification performance was highly significant.

We compared this result to an alternative approach in which the classifier operated on estimates of functional connectivity (Craddock et al., 2009) rather than posterior means of effective connectivity estimates. Following standard practice, functional connectivity was computed in terms of Pearson correlation coefficients among the same eigenvariates of BOLD time series which had been used for DCM. This approach yielded a classification accuracy of 62%. While this was still significantly above chance (*p* < 0.05; infraliminal probability), it was significantly outperformed (*p* < 0.01; Wald test) by generative embedding. Finally, we also tested to what extent classification was possible on the basis of activity levels in the three regions, as encoded by three regional means and three regional standard deviations of GLM parameter estimates. This variant resulted in an accuracy of only 55%, indistinguishable from chance performance (*p* = 0.15; i.p.) and significantly worse (*p* < 0.001; Wald test) than model-based classification.

These results concur with our earlier findings for model-based classification of stroke patients (Brodersen et al., 2011a,b) in the sense that a reasonably good generative model can lead to significantly improved classification performance compared to classification procedures based on descriptive indices such as functional connectivity or regional activity. We discuss the reasons for this superiority of the model-based approach in detail in the Discussion section.

Above and beyond classification accuracy, an advantage of a model-based analysis is that its results can be interpreted in the context of the underlying generative model. To illustrate this, we asked which parameters were most powerful in distinguishing between patients and healthy controls. A simple way of addressing this question is by means of two-sample *t*-tests on individual model parameters (while correcting for multiple tests). This analysis showed that the self-connections of all three regions were significantly discriminative, as was the effective influence exerted by VC onto dLPFC and the connections between dLPFC and PC in either direction (Fig. 4b; *p* < 0.05; Bonferroni-corrected for multiple tests). By contrast, parameters such as the strength of the stimulus input (*C* vector) were not found to be significantly discriminative.

The above supervised (classification) analyses are interesting for comparative purposes and were conducted to verify that our fMRI data and chosen model contain meaningful information about disease state at all. However, we would like to reiterate that these classification analyses are not of primary interest for our present study. Rather than trying to replicate known diagnostic labels based on DSM-IV by fMRI and modelling (which has little priority given the ease with which schizophrenia is diagnosed clinically and the limited predictive validity of DSM categories; see Introduction), the key question we were interested in was whether the schizophrenia spectrum defined by DSM-IV can be dissected into patient subgroups characterised by distinct neurophysiological mechanisms, as encoded by the parameter estimates of our DCM of working memory. This question was addressed by the unsupervised (clustering) analyses presented in the following sections.

### 3.2 Model-based clustering of all subjects

Using the GMM algorithm described in the Methods section, we initially performed a model-based clustering analysis of all subjects, based on their posterior DCM parameter estimates. The purpose of this initial unsupervised analysis was to assess whether our model would be sufficiently sensitive to detect the difference between patients and healthy controls, in the absence of any a priori knowledge about the existence of these two groups. Indeed, we found that the highest model evidence was obtained by a Gaussian mixture model with two clusters (Fig. 4c). This model outperformed the next-best model by a log Bayes factor (BF) of 64.3, implying very strong evidence that a model with two clusters provided the best explanation of the variability observed across subjects.

Evaluating the validity of the obtained clustering solution against the known diagnostic labels (i.e., healthy vs. diagnosed) yielded a balanced purity of 71% (Fig. 4c). That is, the unsupervised assignment of subjects to patient and control groups was almost as good as in the supervised case which had access to considerably more information (namely, that two groups existed, and which subject belonged to which).

To evaluate to what extent the two approaches agreed with regard to individual subjects, we compared the set of subjects that were misclassified to the set of subjects that were ‘misclustered’, in the sense of representing the minority class in their cluster. As expected, the two sets overlapped to a large extent. Specifically, whether or not a subject was classified correctly was predictive, with a balanced accuracy of 83%, of whether he or she was also misclustered. This agreement was much higher than what would be expected by chance (posterior infraliminal probability *p* < 0.001). We found no indication that misclustering was simply a result of ill-fitting models; there was no significant difference in *R*^2^ between correctly and incorrectly clustered patients (*p* = 0.177).

The above model-based classification and clustering results are reassuring, but they ultimately only provide a confirmation of a diagnostic category that is already known and that can be assessed much easier by means of a conventional clinical interview. Instead, the real question of interest is whether the schizophrenic spectrum as defined by DSM-IV may contain neurophysiologically distinct subgroups that are revealed by a model-based characterisation. This motivated the final analysis in which we focussed on the group of patients alone to avoid that more subtle differences amongst the patients could be swamped by more pronounced differences between patients and controls.

### 3.3 Model-based clustering of patients

**Fig. 5:**
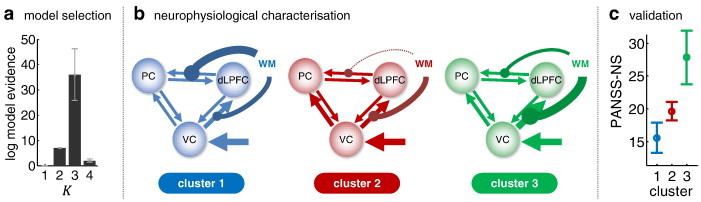
Model-based clustering (generative embedding) of patients. This figure summarises the results of an unsupervised clustering analysis of the patient group only, using Gaussian mixture models operating on DCM parameter estimates. Panel a plots the log evidence for models assuming different numbers of clusters, showing that there is highest evidence for three subgroups of schizophrenic patients. Panel b summarises the average posterior parameter estimates (in terms of *maximum a posteriori* estimates) for each coupling and input parameter in the model. This is displayed graphically by the width of the respective arrows. Panel c demonstrates that the three subgroups, which are defined on the basis of connection strengths, also differ in terms of negative clinical symptoms as operationalised by the negative symptoms (NS) subscale of the PANSS score.

Applying model-based clustering exclusively to the group of participants diagnosed with schizophrenia, we obtained the highest model evidence for a clustering solution with three clusters (Fig. 5a), and model comparison provided very strong evidence that this solution was better than the next-best model, which contained only two clusters (log BF = 29.1). This finding indicates that removing the healthy controls from the analysis unmasked a more subtle distinction among patients, dissecting the schizophrenic spectrum into three distinct clusters.

The identified clusters comprised 22% (*n* = 9), 59% (*n* = 24), and 17% (*n* = 7) of patients, respectively. Critically, since they were defined in terms of a DCM-based generative score space, differences between clusters can be examined in terms of the neuronal circuit models they imply. Fig. 5b illustrates graphically the mean parameter estimates across all subjects within each of the three clusters (see Table 1 for details). This juxtaposition highlights that the three identified subgroups of patients differed in terms of the mechanisms embodied by our model.

Perhaps most striking were the different patterns of modulatory influence exerted by increasing demands on working memory across the three subgroups. Specifically, both the first and the third subgroup showed a strengthening of the visual–prefrontal and the prefrontal–parietal connection under increasing working-memory demands, but the relative strength of these modulatory effects was reversed: working memory primarily enhanced the prefrontal–parietal connection in the first subgroup and the visual–prefrontal connection in the third subgroup. By contrast, the second subgroup showed a weakening of prefrontal–parietal connectivity under increasing working-memory demands and a concomitant increase in the visual–prefrontal connection. This suggests that working memory leads to tighter functional integration of the three areas in the first and third subgroup while causing a relative re-routing of visual information to the prefrontal cortex and a relative decoupling of parietal from prefrontal cortex in the second subgroup.

### 3.4 Validation of the clustering solution

Finally, we evaluated the construct validity of the identified subgroups. In other words, we tested whether the neurophysiologically defined subgroups proposed by our model-based clustering solution could be mapped onto variations in known clinical variables. For this data set, we had access to two clinical variables of interest: (i) *chlorpromazine equivalents* (CPZ) as a measure of medication (where higher values correspond to stronger doses); and (ii) scores on the *positive and negative syndrome scale* (Kay et al., 1987) as a measure of symptom severity in patients with schizophrenia (where higher values correspond to more severe symptoms).

Carrying out a one-way ANOVA separately for these two variables, no significant differences in CPZ equivalents were found among subgroups (*p* = 0.96). By contrast, PANSS scores differed between the subgroups. Specifically, we found significant differences in the ‘negative symptoms’ (NS) subscale (*p* < 0.05). As shown in Fig. 5c, patients in the three clusters scored 15.6 ± 2.3, 19.6 ± 1.4, and 27.9 ± 4.1 (mean ± SD) on this scale. In other words, patients in the second cluster showed an average negative symptom severity that was close to the overall mean in the group of patients; whereas patients in the first and third cluster tended to score below and above this group average, respectively. One could ask (as did one of our reviewers) whether this relation between DCM parameter estimates and PANSS-NS scores can also be found in a supervised analysis. Indeed, a multiple linear regression analysis (with PANSS-NS scores as dependent and DCM parameter estimates as independent variables) across all 40 patients with PANSS scores confirmed the statistical relationship between model parameter estimates and external labels (*p* = 0.008; *F* = 3.02 on 12 and 27 d.f.; *R*2 = 57.3%).

The above relation between the functional architecture of our three-region circuit and a specific PANSS subscale is intriguing and fits nicely with recent reports demonstrating a systematic relationship between working memory performance and severity of negative symptoms. For example, neuropsychological studies by Pantelis et al. (2001) and Pantelis et al. (2004) demonstrated a significant inverse relation between (spatial) working memory performance and the degree of negative symptoms. More recently, the same relationship was also demonstrated for the *n*-back task, as used in our study, by Barr et al. (2010). Finally, in a longitudinal study of first-episode schizophrenic patients, González-Ortega et al. (2013) showed that initial working memory performance predicted the degree of negative symptoms after a 5-year follow-up. The mapping of neurophysiologically informed subgroups based on our three-region working-memory network onto clinical subgroups that differ in negative symptom severity may therefore be a promising target for longitudinal studies, especially given that conventional treatment strategies have largely failed to reduce working-memory deficits and negative symptoms (Hyman and Fenton, 2003; Lieberman et al., 2005).

Given the link between working-memory performance and negative symptoms, one might wonder whether our neurophysiologically defined subgroups simply represented patients that differed in working memory capacity. To test whether the correspondence between our DCM-based clustering results and the PANSS-NS scores could be explained away by differences in working-memory, we carried out another ANOVA based on the difference between 2-back and 0-back working-memory performance. We found no significant differences between clusters (*p* = 0.144).

**Fig. 6:**
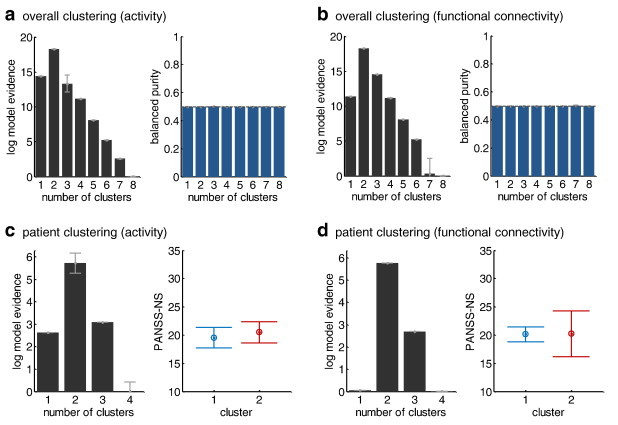
Clustering solutions based on regional activity or functional connectivity. Panels (a) and (b) report the results of repeating the unsupervised clustering analysis of all subjects (cf. Fig. 4c), using estimates of regional activity and functional connectivity, respectively. In contrast to the procedure based on generative embedding, using regional activity and functional connectivity as data features provided weaker relative evidence for two clusters, and these two clusters did not map onto patients and controls (balanced purity insignificantly different from 0.5). Panels (c) and (d) show the results of a clustering analysis of patients only (cf. Fig. 5), using regional activity and functional connectivity. In contrast to the generative-embedding solution, these analyses suggest the existence of two (as opposed to three) patient subgroups. However, in neither case did the suggested subgroups map onto significant differences in clinical symptoms as measured by the PANSS score.

For completeness and comparison, we repeated all clustering analyses for the two feature sets which had proven less powerful than DCM parameter estimates for distinguishing between patients and controls in our initial supervised classification analysis, i.e., regional activity and functional connectivity. The results are summarised in Fig. 6. In brief, GMM-based clustering of all subjects, using either regional activity or functional connectivity, indicated the presence of two groups, as was the case when using effective connectivity. However, the relative evidence for two, compared to fewer or more clusters, was considerably weaker than in our original analysis on the basis of DCM parameters (see the profile of log evidences in Figs. 4c and 6a,b). Accordingly, as one might expect, the two clusters did not map onto patients and controls, as indicated by a balanced purity insignificantly different from 0.5 (Fig. 6a,b). Furthermore, clustering of the patients only led to two subgroups (Fig. 6c,d). Again, however, the relative evidence for this number of subgroups was considerably weaker than in the original model-based analysis (Fig. 5a); and the identified subgroups did not map onto significant differences in clinical symptoms (*p* = 0.73 and *p* = 0.98, respectively; one-way ANOVA; Fig. 6c,d).

## 4 Discussion

In this proof-of-concept study, we extended the recently introduced generative-embedding approach for model-based classification to the unsupervised domain, with a particular focus on the question of how model-based clustering could be used to dissect psychiatric spectrum diseases into physiologically defined subgroups. Using a recently published fMRI dataset of schizophrenic patients and healthy controls performing an *n*-back working-memory task (Deserno et al., 2012), we found that generative embedding, based on a simple three-region DCM of prefrontal-parietal-visual areas, led to significantly better results for both supervised learning (classification) and unsupervised learning (clustering), compared to estimates of functional connectivity or regional activity.

When applied to the patient group alone, generative embedding suggested the existence of three schizophrenic subgroups which were distinguished by differences in network architecture, i.e., the pattern of effective connectivity among prefrontal, parietal, and visual areas. Importantly, even though the definition of these clusters was not informed by any information on clinical symptoms but only by the fMRI data, the three subgroups showed significantly different average PANSS scores (Fig. 5). In other words, our unsupervised model-based clustering procedure identified three neurophysiologically distinct subgroups which mapped onto clinical status. No such correspondence between neurophysiology and symptoms was found when using either functional connectivity estimates or regional activity for clustering (Fig. 6).

One may think that a trivial reason for the superiority of the model-based approach in this application is access to more information, i.e., the analyses based on effective connectivity are based on more features than the analyses based on functional connectivity (posterior means of parameter estimates vs. correlation coefficients). However, this overlooks that in our embedding approach the choice of feature space is a direct consequence of the different assumptions (made by DCM and functional connectivity analyses, respectively) about what constitutes an appropriate low-dimensional summary or sufficient statistics of a network. While DCM posits that a minimal network description includes directed inter-regional influences and their modulation by external perturbations (e.g., task conditions), functional connectivity (as implemented here) only cares about undirected coupling and rests on pair-wise correlation coefficients. Second, and perhaps even more importantly, more data features are not necessarily better: the generalizability of classification and clustering results often starts to decline as the number of features increases, due to overfitting. Indeed, the third type of analysis considered here, based on regional activity was based on twice as many features as functional connectivity, but performed less well (Fig. 4a). Finally, as we demonstrated in previous work (Brodersen et al., 2011a,b), the superiority of a model-based approach to classification critically depends on the quality of its assumptions: in the absence of a good model (or when deliberately compromising model quality), a simpler summary of network dynamics in terms of functional connectivity may be more appropriate and lead to better classification results. In summary, the classification/clustering performance differences of the three different approaches considered here cannot be reduced to differences in the dimensionality of feature spaces; instead, they critically depend on how this feature space was constructed, based on different notions about what constitutes the sufficient statistics of a network.

This superiority of the model-based approach is in accordance with a previous classification study by Brodersen et al. (2011a,b) where generative embedding based on DCM significantly outperformed classification based on functional connectivity or various aspects of regional activity. One plausible reason for this consistent finding is that estimates of effective connectivity are more robust to noise, and variability thereof across regions, than functional connectivity estimates (for a detailed discussion of this issue, see Friston, 2011). Generally, a generative model like DCM separates the measured time series into a predicted component (which is encoded by the model parameters) and unexplained residuals (which represents an estimate of the noise). By harvesting parameter estimates and using these to construct a generative score space, we therefore discard estimates of unexplained noise and restrict classification to those parts of the signal which have a mechanistic explanation (under the assumptions of the model). In other words, by exploiting the very specific denoising effect of fitting a mechanistic model, classification and clustering operate in a more meaningful space and may be more resilient against noise and irrelevant features.

In this study, we have presented a simple generative model of effective connectivity between three regions whose parameters defined patient subgroups which mapped nicely onto a differences in symptom severity. We do not wish to claim that this is the best neurophysiological model one could have devised in order to dissect the spectrum of our schizophrenic patients. Clearly, it would have been possible to consider other mathematical formulations and variants or extensions of the circuit we have considered here. Indeed, in addition to Deserno et al. (2012), numerous other studies have reported connectivity differences during working memory between schizophrenia and healthy controls (Cole et al., 2011; He et al., 2012; Kim et al., 2009; Meda et al., 2009; Rasetti et al., 2011; Repovš and Barch, 2012; Yoon et al., 2013), with some studies highlighting additional brain regions such as the hippocampus or basal ganglia. Here, we focused on the interactions between early visual, parietal, and prefrontal regions; this choice was guided by the voxel-wise analyses by Deserno et al. (2012) for the same data and additionally motivated by the recent meta-analysis of Rottschy et al. (2012) which had emphasised the importance of dorsolateral prefrontal and parietal cortex for working memory.

Which particular model should be preferred for suggesting a neurophysiologically inspired classification can be evaluated with regard to its predictive validity for clinical decision making. Such an evaluation involves testing the suggested classification against clinical benchmarks in longitudinal studies, such as the degree to which the model can predict individual outcome and individual treatment response (see below). More generally, generative embedding suggests an alternative to Bayesian model selection (BMS) for deciding which of several candidate model fits best. While BMS evaluates the evidence of competing models against the same data, generative embedding would rank models in terms of their predictive validity against an external criterion. Both of these approaches have advantages and limitations which are discussed in more detail by Brodersen et al. (2011a,b).

While our study has provided a concrete example that the concept of generative embedding may be useful for splitting psychiatric spectrum diseases into neurophysiologically defined subgroups, it has a number of important limitations. Perhaps most important is the lack of a longitudinal study design which would allow us to test the proposed cluster structure against future clinical variables. For example, it would be convincing and of considerable clinical utility if one found that model-derived neurophysiological subgroups mapped onto treatment-response profiles such as, for example, differential sensitivity to alternative medication options. In our cross-sectional design, we were unable to validate the proposed subgroups with regard to such variables (although we were able to rule out that patients assigned to the three subgroups had significantly different medication in terms of chlorpromazine equivalents).

A second limitation of this study is the type of model used for generative embedding, i.e., a deterministic bilinear DCM for fMRI, which can only provide a rather coarse summary of neurophysiological mechanisms in terms of synaptic coupling between large, undifferentiated neuronal populations. This restriction in conceptual resolution is mainly due to the nature of the fMRI signal which makes it difficult to infer on detailed synaptic mechanisms, such as the contribution of different neurotransmitter systems. Having said this, a number of fMRI studies have demonstrated that, despite the limitations of the BOLD signal, surprisingly precise predictions with clinical utility can be made using DCM, such as inferring the driver of epileptic activity (David et al., 2008) or recognising the presence or absence of dopaminergic medication in patients with Parkinson's disease (Rowe et al., 2010).

Beyond fMRI, even more detailed synaptic mechanisms, such as spike-frequency adaptation or conduction delays, can potentially be inferred by suitably defined DCMs operating on electrophysiological data. For example, in pharmacological studies in both rodents and humans, dynamic causal modelling of the conventional neural mass model type was able to recognise anaesthesia-induced shifts in the balance of excitatory and inhibitory synaptic signalling (Moran et al., 2011a,b). Furthermore, a conductance-based DCM of a prefrontal cortical column succeeded in detecting experimentally induced influences of dopamine on NMDA and AMPA receptor-mediated postsynaptic responses at glutamatergic synapses (Moran et al., 2011a,b). The latter model offers intriguing opportunities for future model-based prediction studies in schizophrenia given that it aims to quantify a physiological process (i.e., dopaminergic influence on glutamatergic signalling) which possesses a direct link to both pathophysiological theories (cf. the dis-/dysconnection hypothesis; Friston, 1998; Stephan et al., 2009) and treatment options in schizophrenia. Another promising extension may be to construct model-based analyses that rest on a combination of dynamical system models of neurophysiology with computational models of behaviour (e.g., Den Ouden et al., 2010). An example of behavioural models that are also of a generative nature and therefore suitable for such a combined approach are meta-Bayesian models of learning and decision making (e.g., Daunizeau et al., 2010; Mathys et al., 2011). This combined approach has great potential for enabling what one might refer to as *neurocomputational generative embedding*, affording classification and clustering results with both behavioural and neurophysiological interpretability.

It is worth emphasising that this paper should not be misunderstood as arguing for a purely categorical classification and against a dimensional construct of psychiatric diseases. While this is a complicated issue with a long history (cf. Allardyce et al., 2007; Cloninger et al., 1985; Demjaha et al., 2009; Everitt et al., 1971) which we cannot cover in detail here, we would like to emphasise that under any perspective that postulates a mapping from the state of neuronal circuits to phenomenology, categorical and dimensional perspectives naturally coexist and complement each other. For example, at the circuit level represented by the type of model used in this study, each parameter (such as those encoding the strength of a particular synaptic connection) lives on a continuous scale, and thus any particular aspect of the patient's symptoms affected by this particular parameter is best understood as a dimensional construct. Nevertheless, within the space spanned by the circuit's parameters, certain configurations (parameter vectors) may occur more frequently across subjects than others, thus forming clusters which can be understood as neurophysiologically defined categories of the disease state overall. In general, subgroups like those found in the present study should be scrutinized by future longitudinal studies, specifically designed to test for differences in clinically meaningful outcome variables, such as response to a particular treatment. This could provide a useful strategy towards a revised nosology based on mechanistic accounts with concrete treatment implications, irrespective of whether one prefers a dimensional or categorical perspective on the underlying model.

Regarding the statistical approach chosen in the present study, one can imagine an alternative strategy that unifies mechanistically inspired modelling of the observed data and classification or clustering within a single generative model. This would require a model describing how clinical outcomes depend on hidden (latent) neurophysiological mechanisms which themselves depend on underlying causes (disease subgroups) whose cardinality is unknown and must be inferred, either by Bayesian model selection (in the same way as was done in the case of a GMM in the present paper) or, by adopting a nonparametric Bayesian approach, jointly with the parameters of the neurophysiological model. Such potential extensions are the topic of ongoing work in our group. Unified generative models of this sort could reduce the two steps of generative embedding and classification or clustering (steps 3 and 4 in Fig. 1) to a single step of model inversion. On the other hand, similar to group analyses in which a second-level test on first-level summary statistics often provides a close approximation to full hierarchical mixed-effects models (Brodersen et al., 2012; Mumford and Nichols, 2009), it could turn out that generative embedding is simpler and more robust than inverting the unified generative model sketched out above. Whichever statistical machinery is chosen, given the promising potential shown by its initial applications to classification (Brodersen et al., 2011a,b) and clustering (this study), we anticipate that model-based analyses of this sort will serve as a useful framework for future applications of machine learning and computational neuroscience to psychiatry.

## Figures and Tables

**Table 1:** Connectivity within the three identified patient clusters. The table summarizes the connectivity of the three clusters identified by Gaussian mixture modelling of the 41 individual dynamic causal models of patients with schizophrenia. Each row shows a particular model parameter. All parameter estimates are given in terms of mean ± standard error across the patients contained in each cluster. For a graphical interpretation of the means, see Fig. 5b.

Model parameter	Cluster 1 (*n* = 9)	Cluster 2 (*n* = 24)	Cluster 3 (*n* = 7)
VC self-connection	− 1.000 ± 0.001	− 0.994 ± 0.003	− 1.001 ± 0.004
VC → PC	0.036 ± 0.022	0.070 ± 0.036	0.044 ± 0.025
VC → DLPFC	0.055 ± 0.029	0.044 ± 0.018	0.130 ± 0.068
PC → VC	0.017 ± 0.001	0.014 ± 0.004	0.017 ± 0.002
PC self-connection	− 1.000 ± 0.000	− 0.994 ± 0.004	− 0.999 ± 0.001
PC → DLPFC	0.017 ± 0.001	0.014 ± 0.005	0.024 ± 0.007
DLPFC → VC	0.043 ± 0.026	0.021 ± 0.02	0.019 ± 0.004
DLPFC → PC	0.042 ± 0.024	0.034 ± 0.012	0.023 ± 0.008
DLPFC self-connection	− 0.997 ± 0.003	− 0.998 ± 0.001	− 0.996 ± 0.003
WM modulation of VC → DLPFC	0.110 ± 0.227	0.124 ± 0.102	0.267 ± 0.135
WM modulation of DLPFC → PC	0.204 ± 0.191	− 0.023 ± 0.017	0.071 ± 0.064
Direct input to VC	0.073 ± 0.043	0.074 ± 0.043	0.104 ± 0.020

## Appendix A

Variational Gaussian mixture model

This section details the prior parameterization we use to obtain a variational Bayesian solution to the Gaussian mixture model used in the main text. To begin with, we define Wishart priors on the precision matrices *Λ* and Gaussian priors on the means *μ* (conveniently parameterised as conditional priors given the respective prior precisions). Further, we define a diffuse Dirichlet prior over the mixing coefficients *π*. In summary:

(1) $$p\left(\Lambda \right)=\prod_{k=1}^{K}W\left(\Lambda_{k}|W_{0},\nu_{0}\right)$$

(2) $$p\left(\mu |\Lambda \right)=\prod_{k=1}^{K}N\left(\mu_{k}|m_{o},{\left(\beta_{0}\Lambda_{k}\right)}^{-1}\right)$$

(3) $$p\left(\pi \right)=\mathrm{Dir}\left(\pi |\alpha_{0}\right).$$

Using this parameterisation, the only approximating assumption required to obtain a variational solution is the mean-field assumption

(4) $$q\left(Z,\pi ,\mu ,\Lambda \right)=q\left(Z\right)q\left(\pi ,\mu ,\Lambda \right),$$

where *q*(⋅) is the desired variational approximation to the true posterior *p*(⋅|*X*). To find the optimal variational posterior *q**(⋅), the algorithm iterates between a variational E-step,

(5) $$\ln q^{*}\left(Z\right)={\langle \ln p\left(X,Z,\pi ,\mu ,\Lambda \right)\rangle }_{q\left(\pi ,\mu ,\Lambda \right)},$$

and a variational M-step,

(6) $$\ln q^{*}\left(\pi ,\mu ,\Lambda \right)={\langle \ln p\left(X,Z,\pi ,\mu ,\Lambda \right)\rangle }_{q\left(Z\right)}.$$

Critically, this approach enables us to determine the optimal number of clusters by means of Bayesian model selection. Specifically, we can compute a *free-energy* bound to the log model evidence

(7) $$\begin{array}{l} \ln p\left(X\right)=\ln∭p\left(X|Z,\pi ,\mu ,\Lambda \right)p\left(Z,\pi ,\mu ,\Lambda \right)dZd\pi d\mu d\Lambda \\ \;=\mathrm{KL}\left[q∥p\right]+F\left(q,X\right)\\ \;\geq {\langle \ln p\left(X,Z,\pi ,\mu ,\Lambda \right)\rangle }_{q}+H\left[q\right]. \end{array}$$

In the above equation, we have expressed the log model evidence as the sum of: the Kullback–Leibler divergence *KL*[*q* ∥ *p*] between the approximate posterior *q* = *q*(*Z*,*π*,*μ*,Λ) and the true posterior *p* ≡ *p*(*Z*, *π*, *μ*, Λ|*X*); and the negative free energy *F*(*q*,*X*). Maximising the negative free energy minimises the KL-divergence and thus leads to a maximally close approximation to the true posterior. The free energy itself can be easily evaluated for a given *q* as the sum of: the expected log joint distribution over the data and the parameters 〈 ln *p*(*X*,*Z*,*π*,*μ*,Λ〉_*q*_; and the Shannon entropy of the approximate posterior, *H*[*q*].

## References

[bb0005] Allardyce J., McCreadie R.G., Morrison G., van Os J. (2007). Do symptom dimensions or categorical diagnoses best discriminate between known risk factors for psychosis?. Soc. Psychiatry Psychiatr. Epidemiol..

[bb0010] Attias H. (2000). A variational Bayesian framework for graphical models. Adv. Neural Inf. Process. Syst..

[bb0015] Barch D.M., Carter C.S., Dakin S.C., Gold J., Luck S.J., MacDonald A., Strauss M.E. (2012). The clinical translation of a measure of gain control: the contrast–contrast effect task. Schizophr. Bull..

[bb0020] Barr M.S., Farzan F., Tran L.C., Chen R., Fitzgerald P.B., Daskalakis Z.J. (2010). Evidence for excessive frontal evoked gamma oscillatory activity in schizophrenia during working memory. Schizophr. Res..

[bb0025] Bishop C.M. (2007). Pattern Recognition and Machine Learning.

[bb0030] Borgwardt S., Radua J., Mechelli A., Fusar-Poli P. (2012). Why are psychiatric imaging methods clinically unreliable? Conclusions and practical guidelines for authors, editors and reviewers. Behav. Brain Funct..

[bb0035] Bowie C.R., Leung W.W., Reichenberg A., McClure M.M., Patterson T.L., Heaton R.K., Harvey P.D. (2008). Predicting schizophrenia patients' real-world behavior with specific neuropsychological and functional capacity measures. Biol. Psychiatry.

[bb0040] Braff D.L., Freedman R. (2008). Clinically responsible genetic testing in neuropsychiatric patients: a bridge too far and too soon. Am. J. Psychiatry.

[bb0060] Brodersen K.H., Ong C.S., Stephan K.E., Buhmann J.M. (2010). The balanced accuracy and its posterior distribution. *Proceedings of the 20th International Conference on Pattern Recognition* Presented at the International Conference on Pattern Recognition.

[bb0050] Brodersen K.H., Haiss F., Ong C.S., Jung F., Tittgemeyer M., Buhmann J.M., Stephan K.E. (2011). Model-based feature construction for multivariate decoding. NeuroImage.

[bb0065] Brodersen K.H., Schofield T.M., Leff A.P., Ong C.S., Lomakina E.I., Buhmann J.M., Stephan K.E. (2011). Generative embedding for model-based classification of fMRI data. PLoS Comput. Biol..

[bb0055] Brodersen K.H., Mathys C., Chumbley J.R., Daunizeau J., Ong C.S., Buhmann J.M., Stephan K.E. (2012). Bayesian mixed-effects inference on classification performance in hierarchical data sets. J. Mach. Learn. Res..

[bb0045] Brodersen K.H., Daunizeau J., Mathys C., Chumbley J.R., Buhmann J.M., Stephan K.E. (2013). Variational Bayesian mixed-effects inference for classification studies. NeuroImage.

[bb0070] Caspi A., Moffitt T.E. (2006). Gene-environment interactions in psychiatry: joining forces with neuroscience. Nat. Rev. Neurosci..

[bb0075] Chang C.-C., Lin C.-J. (2011). LIBSVM: a library for support vector machines. ACM Trans. Intell. Syst. Technol..

[bb0080] Chen C.C., Kiebel S.J., Friston K.J. (2008). Dynamic causal modelling of induced responses. NeuroImage.

[bb0085] Cloninger C.R., Martin R.L., Guze S.B., Clayton P.J. (1985). Diagnosis and prognosis in schizophrenia. Arch. Gen. Psychiatry.

[bb0090] Cole M.W., Anticevic A., Repovs G., Barch D. (2011). Variable global dysconnectivity and individual differences in schizophrenia. Biol. Psychiatry.

[bb0095] Collignon O., Dormal G., Albouy G., Vandewalle G., Voss P., Phillips C., Lepore F. (2013). Impact of blindness onset on the functional organization and the connectivity of the occipital cortex. Brain.

[bb0100] Costafreda S.G., Khanna A., Mourao-Miranda J., Fu C.H.Y. (2009). Neural correlates of sad faces predict clinical remission to cognitive behavioural therapy in depression. Neuroreport.

[bb0105] Craddock R.C., P. E. H., Hu X.P., Mayberg H.S. (2009). Disease state prediction from resting state functional connectivity. Magn. Reson. Med..

[bb9000] Cross-Disorder Group of the Psychiatric Genomics Consortium (2013). Identification of risk loci with shared effects on five major psychiatric disorders: a genome-wide analysis. Lancet.

[bb0115] Daunizeau J., Kiebel S.J., Friston K.J. (2009). Dynamic causal modelling of distributed electromagnetic responses. NeuroImage.

[bb0110] Daunizeau J., den Ouden H.E.M., Pessiglione M., Kiebel S.J., Stephan K.E., Friston K.J. (2010). Observing the observer (I): meta-Bayesian models of learning and decision-making. PLoS ONE.

[bb0120] Davatzikos C., Fan Y., Wu X., Shen D., Resnick S.M. (2008). Detection of prodromal Alzheimer's disease via pattern classification of magnetic resonance imaging. Neurobiol. Aging.

[bb0130] David O., Kiebel S.J., Harrison L.M., Mattout J., Kilner J.M., Friston K.J. (2006). Dynamic causal modeling of evoked responses in EEG and MEG. NeuroImage.

[bb0125] David O., Guillemain I., Saillet S., Reyt S., Deransart C., Segebarth C., Depaulis A. (2008). Identifying neural drivers with functional MRI: an electrophysiological validation. PLoS Biol..

[bb0135] Demjaha A., Morgan K., Morgan C., Landau S., Dean K., Reichenberg A., Dazzan P. (2009). Combining dimensional and categorical representation of psychosis: the way forward for DSM-V and ICD-11?. Psychol. Med..

[bb0140] Dempster E.L., Pidsley R., Schalkwyk L.C., Owens S., Georgiades A., Kane F., Mill J. (2011). Disease-associated epigenetic changes in monozygotic twins discordant for schizophrenia and bipolar disorder. Hum. Mol. Genet..

[bb0145] Den Ouden H.E.M., Daunizeau J., Roiser J., Friston K.J., Stephan K.E. (2010). Striatal prediction error modulates cortical coupling. J. Neurosci..

[bb0150] Deserno L., Sterzer P., Wüstenberg T., Heinz A., Schlagenhauf F. (2012). Reduced prefrontal–parietal effective connectivity and working memory deficits in schizophrenia. J. Neurosci..

[bb0155] Desseilles M., Schwartz S., Dang-Vu T.T., Sterpenich V., Ansseau M., Maquet P., Phillips C. (2011). Depression alters “top-down” visual attention: a dynamic causal modeling comparison between depressed and healthy subjects. NeuroImage.

[bb0160] Everitt B.S., Gourlay A.J., Kendell R.E. (1971). An attempt at validation of traditional psychiatric syndromes by cluster analysis. Br. J. Psychiatry J. Ment. Sci..

[bb0165] Friston K.J. (1998). The disconnection hypothesis. Schizophr. Res..

[bb0185] Friston Karl J. (2011). Functional and effective connectivity: a review. Brain Connectivity.

[bb0175] Friston K.J., Harrison L., Penny W. (2003). Dynamic causal modelling. NeuroImage.

[bb0180] Friston K., Mattout J., Trujillo-Barreto N., Ashburner J., Penny W. (2006). Variational free energy and the Laplace approximation. NeuroImage.

[bb0190] Fusar-Poli P., Bonoldi I., Yung A.R. (2012). Predicting psychosis: meta-analysis of transition outcomes in individuals at high clinical risk. Arch. Gen. Psychiatry.

[bb0195] González-Ortega I., de Los Mozos V., Echeburúa E., Mezo M., Besga A., Ruiz de Azúa S., González-Pinto A. (2013). Working memory as a predictor of negative symptoms and functional outcome in first episode psychosis. Psychiatry Res..

[bb0200] He H., Sui J., Yu Q., Turner J.A., Ho B.-C., Sponheim S.R., Calhoun V.D. (2012). Altered small-world brain networks in schizophrenia patients during working memory performance. PLoS ONE.

[bb0205] Hyman S.E., Fenton W.S. (2003). Medicine. What are the right targets for psychopharmacology?. Science (New York, N.Y.).

[bb0215] Insel T., Cuthbert B., Garvey M., Heinssen R., Pine D.S., Quinn K., Wang P. (2010). Research domain criteria (RDoC): toward a new classification framework for research on mental disorders. Am. J. Psychiatry.

[bb0220] International Schizophrenia Consortium, Purcell S.M., Wray N.R., Stone J.L., Visscher P.M., O'Donovan M.C., Sklar P. (2009). Common polygenic variation contributes to risk of schizophrenia and bipolar disorder. Nature.

[bb0225] Johnstone E.C., Crow T.J., Frith C.D., Owens D.G. (1988). The Northwick Park “functional” psychosis study: diagnosis and treatment response. Lancet.

[bb0230] Johnstone E.C., Frith C.D., Crow T.J., Owens D.G., Done D.J., Baldwin E.J., Charlette A. (1992). The Northwick Park “Functional” Psychosis Study: diagnosis and outcome. Psychol. Med..

[bb0235] Kapur S., Phillips A.G., Insel T.R. (2012). Why has it taken so long for biological psychiatry to develop clinical tests and what to do about it?. Mol. Psychiatry.

[bb0240] Kass R.E., Raftery A.E. (1995). Bayes factors. J. Am. Stat. Assoc..

[bb0245] Kay S.R., Fiszbein A., Opler L.A. (1987). The positive and negative syndrome scale (PANSS) for schizophrenia. Schizophr. Bull..

[bb0250] Kim D.I., Manoach D.S., Mathalon D.H., Turner J.A., Mannell M., Brown G.G., Calhoun V.D. (2009). Dysregulation of working memory and default-mode networks in schizophrenia using independent component analysis, an fBIRN and MCIC study. Hum. Brain Mapp..

[bb0255] Klöppel S., Abdulkadir A., Jack C.R., Koutsouleris N., Mourão-Miranda J., Vemuri P. (2012). Diagnostic neuroimaging across diseases. NeuroImage.

[bb0260] Koutsouleris N., Borgwardt S., Meisenzahl E.M., Bottlender R., Möller H.-J., Riecher-Rössler A. (2012). Disease prediction in the at-risk mental state for psychosis using neuroanatomical biomarkers: results from the FePsy study. Schizophr. Bull..

[bb0265] Lasserre J.A., Bishop C.M., Minka T.P. (2006). Principled hybrids of generative and discriminative models.

[bb0270] Lee J., Park S. (2005). Working memory impairments in schizophrenia: a meta-analysis. J. Abnorm. Psychol..

[bb0275] Lehmann M., Barnes J., Ridgway G.R., Ryan N.S., Warrington E.K., Crutch S.J., Fox N.C. (2012). Global gray matter changes in posterior cortical atrophy: a serial imaging study. Alzheimers Dement..

[bb0280] Lewis D.A., Gonzalez-Burgos G. (2006). Pathophysiologically based treatment interventions in schizophrenia. Nat. Med..

[bb0285] Lieberman J.A., Stroup T.S., McEvoy J.P., Swartz M.S., Rosenheck R.A., Perkins D.O. (2005). Clinical antipsychotic trials of intervention effectiveness (CATIE) investigators effectiveness of antipsychotic drugs in patients with chronic schizophrenia. N. Engl. J. Med..

[bb0290] Liu F., Guo W., Yu D., Gao Q., Gao K., Xue Z., Chen H. (2012). Classification of different therapeutic responses of major depressive disorder with multivariate pattern analysis method based on structural MR scans. PLoS ONE.

[bb0295] MacKay D.J. (1992). A practical Bayesian framework for backpropagation networks. Neural Comput..

[bb0300] Manning C.D., Raghavan P., Schütze H. (2008). http://www.muict.polppolservice.com/Year3_2/IR/SEC3/L8%5B4slides%5D.pdf.

[bb0305] Martins A., Bicego M., Murino V., Aguiar P., Figueiredo M. (2010). Information theoretical kernels for generative embeddings based on Hidden Markov Models. Struct. Syntactic Stat. Pattern Recognit..

[bb0310] Mathys C., Daunizeau J., Friston K.J., Stephan K.E. (2011). A Bayesian foundation for individual learning under uncertainty. Front. Hum. Neurosci..

[bb0315] Meda S.A., Stevens M.C., Folley B.S., Calhoun V.D., Pearlson G.D. (2009). Evidence for anomalous network connectivity during working memory encoding in schizophrenia: an ICA based analysis. PLoS ONE.

[bb0320] Minka T. (2005). Discriminative models, not discriminative training (No. MSR-TR-2005-144). Microsoft Research. ftp://ftp.research.microsoft.com/pub/tr/TR-2005-144.pdf.

[bb0325] Moran R.J., Stephan K.E., Seidenbecher T., Pape H.-C., Dolan R.J., Friston K.J. (2009). Dynamic causal models of steady-state responses. NeuroImage.

[bb0330] Moran Rosalyn J., Jung F., Kumagai T., Endepols H., Graf R., Dolan R.J., Tittgemeyer M. (2011). Dynamic causal models and physiological inference: a validation study using isoflurane anaesthesia in rodents. PLoS ONE.

[bb0335] Moran Rosalyn J., Symmonds M., Stephan K.E., Friston K.J., Dolan R.J. (2011). An in vivo assay of synaptic function mediating human cognition. Curr. Biol..

[bb0340] Mourao-Miranda J., Reinders A.A.T.S., Rocha-Rego V., Lappin J., Rondina J., Morgan C., Dazzan P. (2012). Individualized prediction of illness course at the first psychotic episode: a support vector machine MRI study. Psychol. Med..

[bb0345] Mumford J.A., Nichols T. (2009). Simple group fMRI modeling and inference. NeuroImage.

[bb0350] Nuechterlein K.H., Subotnik K.L., Green M.F., Ventura J., Asarnow R.F., Gitlin M.J., Mintz J. (2011). Neurocognitive predictors of work outcome in recent-onset schizophrenia. Schizophr. Bull..

[bb0360] Pantelis C., Stuart G.W., Nelson H.E., Robbins T.W., Barnes T.R. (2001). Spatial working memory deficits in schizophrenia: relationship with tardive dyskinesia and negative symptoms. Am. J. Psychiatry.

[bb0355] Pantelis C., Harvey C.A., Plant G., Fossey E., Maruff P., Stuart G.W., Barnes T.R.E. (2004). Relationship of behavioural and symptomatic syndromes in schizophrenia to spatial working memory and attentional set-shifting ability. Psychol. Med..

[bb0365] Penny W.D., Stephan K.E., Mechelli A., Friston K.J. (2004). Comparing dynamic causal models. NeuroImage.

[bb0370] Perina A., Cristani M., Castellani U., Murino V., Jojic N. (2010). A hybrid generative/discriminative classification framework based on free-energy terms. IEEE 12th International Conference on Computer Vision.

[bb0375] Petronis A., Gottesman I.I., Kan P., Kennedy J.L., Basile V.S., Paterson A.D., Popendikyte V. (2003). Monozygotic twins exhibit numerous epigenetic differences: clues to twin discordance?. Schizophr. Bull..

[bb0380] Rasetti R., Sambataro F., Chen Q., Callicott J.H., Mattay V.S., Weinberger D.R. (2011). Altered cortical network dynamics: a potential intermediate phenotype for schizophrenia and association with ZNF804A. Arch. Gen. Psychiatry.

[bb0385] Repovš G., Barch D.M. (2012). Working memory related brain network connectivity in individuals with schizophrenia and their siblings. Front. Hum. Neurosci..

[bb0390] Rottschy C., Langner R., Dogan I., Reetz K., Laird A.R., Schulz J.B., Eickhoff S.B. (2012). Modelling neural correlates of working memory: a coordinate-based meta-analysis. NeuroImage.

[bb0395] Rowe J.B., Hughes L.E., Barker R.A., Owen A.M. (2010). Dynamic causal modelling of effective connectivity from fMRI: are results reproducible and sensitive to Parkinson's disease and its treatment?. NeuroImage.

[bb0400] Schmidt A., Diaconescu A.O., Kometer M., Friston K.J., Stephan K.E., Vollenweider F.X. (2013). Modeling ketamine effects on synaptic plasticity during the mismatch negativity. Cerebral Cortex.

[bb0405] Shamsi S., Lau A., Lencz T., Burdick K.E., DeRosse P., Brenner R., Malhotra A.K. (2011). Cognitive and symptomatic predictors of functional disability in schizophrenia. Schizophr. Res..

[bb0410] Siegle G., Thompson W. (2012). Toward clinically useful neuroimaging in depression treatment: prognostic utility of subgenual cingulate activity for determining depression outcome in cognitive therapy across studies, scanners, and patient characteristics. Arch. Gen. Psychiatry.

[bb0415] Snitz B.E., MacDonald A.W., Carter C.S. (2006). Cognitive deficits in unaffected first-degree relatives of schizophrenia patients: a meta-analytic review of putative endophenotypes. Schizophr. Bull..

[bb0430] Stephan Klaas Enno (2004). On the role of general system theory for functional neuroimaging. J. Anat..

[bb0440] Stephan Klaas Enno, Weiskopf N., Drysdale P.M., Robinson P.A., Friston K.J. (2007). Comparing hemodynamic models with DCM. NeuroImage.

[bb0435] Stephan Klaas Enno, Kasper L., Harrison L.M., Daunizeau J., den Ouden H.E.M., Breakspear M., Friston K.J. (2008). Nonlinear dynamic causal models for fMRI. NeuroImage.

[bb0425] Stephan Klaas E., Friston K.J., Frith C.D. (2009). Dysconnection in schizophrenia: from abnormal synaptic plasticity to failures of self-monitoring. Schizophr. Bull..

[bb0420] Stephan K.E., Penny W.D., Moran R.J., den Ouden H.E.M., Daunizeau J., Friston K.J. (2010). Ten simple rules for dynamic causal modeling. NeuroImage.

[bb0445] Szeszko P.R., Narr K.L., Phillips O.R., McCormack J., Sevy S., Gunduz-Bruce H., Robinson D.G. (2012). Magnetic resonance imaging predictors of treatment response in first-episode schizophrenia. Schizophr. Bull..

[bb0450] Tansey K.E., Guipponi M., Perroud N., Bondolfi G., Domenici E., Evans D., Uher R. (2012). Genetic predictors of response to serotonergic and noradrenergic antidepressants in major depressive disorder: a genome-wide analysis of individual-level data and a meta-analysis. PLoS Med..

[bb0455] Van Leeuwen T.M., den Ouden H.E.M., Hagoort P. (2011). Effective connectivity determines the nature of subjective experience in grapheme-color synesthesia. J. Neurosci. Off. J. Soc. Neurosci..

[bb0460] Vesterager L., Christensen T.Ø., Olsen B.B., Krarup G., Melau M., Forchhammer H.B., Nordentoft M. (2012). Cognitive and clinical predictors of functional capacity in patients with first episode schizophrenia. Schizophr. Res..

[bb0465] Yoon J.H., Minzenberg M.J., Raouf S., D'Esposito M., Carter C.S. (2013). Impaired prefrontal–basal ganglia functional connectivity and substantia nigra hyperactivity in schizophrenia. Biol. Psychiatry.

